# Acceptability of palliative care approaches for patients with severe and persistent mental illness: a survey of psychiatrists in Switzerland

**DOI:** 10.1186/s12888-019-2091-x

**Published:** 2019-04-11

**Authors:** Manuel Trachsel, Martina A. Hodel, Scott A. Irwin, Paul Hoff, Nikola Biller-Andorno, Florian Riese

**Affiliations:** 1 Institute of Biomedical Ethics and History of Medicine, University of Zurich, Winterthurerstrasse 30, 8006 Zurich, Switzerland; 2 Cedars-Sinai Medical Center, 8700 Beverly Blvd, Los Angeles, CA 90048 USA; 3 Division of Psychiatry Research and Psychogeriatric Medicine, Psychiatric University Hospital Zurich, Lenggstrasse 31, 8008 Zurich, Switzerland; 4 Department of Psychiatry, Psychotherapy and Psychosomatics, Psychiatric University Hospital Zurich, Zurich, Switzerland

**Keywords:** Severe and persistent mental illness, Goals of care, Quality of life, Treatment-refractoriness, Futility, Palliative care, Palliative psychiatry

## Abstract

**Background:**

Some patients develop severe and persistent mental illness (SPMI) which is therapy-refractory. The needs of these patients sometimes remain unmet by therapeutic interventions and they are at high risk of receiving care that is inconsistent with their life goals. Scholarly discourse has recently begun to address the suitability of palliative care approaches targeting at enhancing quality of life for these patients, but remains to be developed.

**Method:**

A cross-sectional survey asked 1311 German-speaking psychiatrists in Switzerland (the total number of German-speaking members of the Swiss Society for Psychiatry and Psychotherapy) about the care of SPMI patients in general, and about palliative care approaches in particular. 457 (34.9%) returned the completed survey. In addition, participants were asked to evaluate three case vignettes of patients with SPMI.

**Results:**

The reduction of suffering and maintaining daily life functioning of the patient were rated as considerably more important in the treatment of SPMI than impeding suicide and curing the underlying illness. There was broad agreement that SPMI can be terminal (93.7%), and that curative approaches may sometimes be futile (e.g. 72.4% for the anorexia nervosa case vignette). Furthermore, more than 75% of the participating psychiatrists were in favour of palliative care approaches for SPMI.

**Conclusions:**

The results of the present study suggest that the participating psychiatrists in Switzerland regard certain forms of SPMI as posing high risk of death. Additionally, a majority of respondents consider palliative care approaches appropriate for this vulnerable group of patients. However, the generalizability of the results to all psychiatrists in Switzerland or other mental health professionals involved in the care of SPMI is limited. This limitation is important considering the reservations towards palliative care in the context of psychiatric illness, mainly because of the association with death and futility. Palliative care approaches, however, are applicable in conjunction with other therapies intended to prolong life. A next step could be to involve service users and develop a consensus of what palliative care might encompass in SPMI. A framework for identifying which patients might benefit from palliative care, should be explored for the future development of care for SPMI patients.

## Background

According to the World Health Organization [1], ‘[p]alliative care is an approach that improves the quality of life of patients and their families facing the problem associated with life-threatening illness, through the prevention and relief of suffering by means of early identification and impeccable assessment and treatment of pain and other problems, physical, psychosocial and spiritual […]’. Based on this broad definition, some psychiatric interventions may be considered palliative, as they aim primarily to enhance quality of life by means of adequate symptom control and by focusing on disability rather than on curing the illness [2]. However, palliative care as a deliberate approach has not been widely implemented in mental healthcare, and its tools have not been deployed in psychiatric practice. Its relevance in the treatment of certain severe and persistent mental illness (SPMI) such as severe and persistent depression, schizophrenia and anorexia nervosa [2–6] has only recently been suggested. Studies have consistently shown higher mortality among patients with SPMI [7, 8], who die 10 to 20 years earlier on average than persons in the general population [9, 10]. At the same time, a large body of research has focused on ultra-high risk and prodromal paradigms, representing a clear emphasis on early interventions perhaps at the cost of the development of adequate psychosocial care for patients in later stages of the disease [11, 12]. Although full remission or recovery is the primary goal of acute psychiatric treatment, a substantial number of patients diagnosed with a major depressive disorder are resistant to evidence-based treatments, including treatments for chronic depression such as electro-convulsive therapy [13] or ketamine infusion [14], and remission rates decrease with each additional treatment trial [15]. In cases of schizophrenia, about one fifth of all patients show little or no therapeutic response and exhibit increased susceptibility to several life-threatening comorbidities [16]. For these patients, evidence-based illness-modifying approaches are unavailable or remain ineffective, leading to low quality of life and frequent use of healthcare services [3]. The contentious scholarly discourse surrounding the application of palliative care approaches centres on the futility debate and is often linked to anecdotal reports, usually in the context of severe anorexia nervosa [3–6, 17–20]. In these circumstances, there is a risk that palliative care approaches in psychiatry may be perceived as inevitably intertwined with ‘giving up’ and losing hope rather than as complementary to recovery-oriented models [21] for specific cases of SPMI. However, it is important to acknowledge that additional experimental treatment trials (e.g. low yield, higher risk polypharmacy) can sometimes leave patients more demoralized as they are caught in a cycle of false hope [3]. For patients with a low probability of a favourable treatment outcome, it is therefore important to develop a modern concept of supportive care that does not ignore or trivialise the catastrophic effect some mental illnesses can have. Such approaches should focus on a psychosocial support system that goes beyond the traditional mindset of psychiatric care. The acceptability of certain palliative care approaches for severe and persistent mentally ill patients whose needs cannot be met by contemporary therapeutic interventions, however, is unclear. Since psychiatrists are the main decision makers when it comes to SPMI patients, it is crucial to initially explore the extent of acceptance of palliative care approaches in treating SPMI patients. We therefore asked practicing physicians with a specialist training in psychiatry (hereinafter: psychiatrists) to evaluate the suitability of such approaches. The main research questions concerned whether psychiatrists in Switzerland considered palliative care approaches to be appropriate for SPMI in general and for certain diagnostic groups in particular, and how they evaluated futility in specific cases. Additionally, we were interested in the prioritization of common treatment goals in cases of SPMI, such as everyday functioning and the reduction of suffering.

## Methods

A quantitative cross-sectional survey was conducted in cooperation with the Swiss Society for Psychiatry and Psychotherapy (SSPP), in accordance with the ethical review processes of the University of Zurich and the checklist for the ethical evaluation of empirical studies.

### Sample

The sample comprised all German-speaking members of the SSPP who are practicing psychiatrists (*n* = 1311), corresponding to approximately 30% of psychiatrists in Switzerland. About 70% of psychiatrists in Switzerland are either French- or Italian-speaking or are not SSPP members. The rationale for sampling solely from members of the SSPP lies in the fact that in Switzerland, reliable contact information of physicians is only available in cooperation with relevant professional organizations such as the SSPP in which membership is not mandatory for psychiatrists. The SSPP contacted all participants prior to the survey to inform them of its purpose. Data were collected in the period February–March 2017.

### Procedure

All participants received a hard copy of the survey with an enclosed prepaid return envelope (paper-pencil format). There was no incentive for participation. Participants also received a reminder postcard four weeks later.

### Survey and case vignettes

**Fig. 1:**
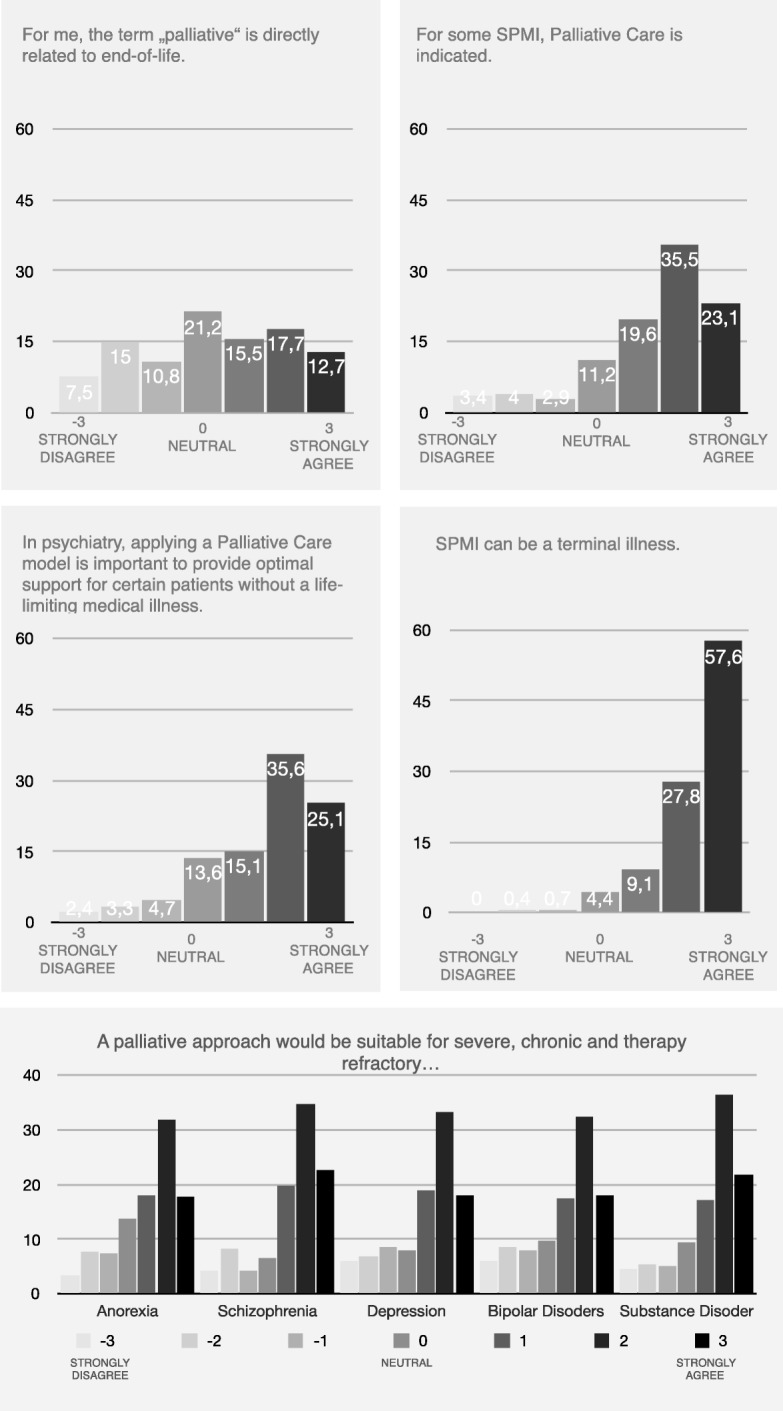
Psychiatrists’ attitudes to palliative care and severe and persistent mental illness

The cross-sectional survey was based on the research questions. The case vignettes drew on previously published material [6, 22, 23] and were adapted to suit the format and goal of the survey (see Table 1). The content of survey items and case vignettes was revised by an advisory group that included experts and trainees in psychiatric practice and/or research, as well as biostatisticians. Participants were asked to respond to 18 items on a 7-point Likert scale, ranging from *completely disagree* (− 3) to completely agree (+ 3), with a neutral mid-point (0), or from *unimportant* (score: 0) to *very important* (6). (See Table 2 for all survey items.) Items related to the three case vignettes (7 questions in each) adopted the same response format. The total number of items (including case vignettes) was 42. The WHO definition of palliative care [1] was also provided (Fig. 1).

**Table 1:** Case vignettes based on modified versions of previously published cases (6,22,23)

Case 1: 37-year-old female with anorexia nervosa, onset at age 11	
Symptoms: general muscle weakness; loss of bone density; amenorrhea; current weight 24 kg/52 lbs.; BMI 9.5 kg/m2; no recent weight gain or stabilization; no acute danger of dying, as her body is adapted to being underweight. The patient underwent 10 previous inpatient treatments (in both somatic and psychiatric hospitals), three of which were in specialized psychiatric institutions. Throughout the course of disease, different intensive psychotherapies have been tried, without success. During hospitalizations, the patient underwent several artificial re-feedings, sometimes under sedation. The patient now refuses artificial re-feeding and treatment. She states that, for years, her life has been focused exclusively on trying to overcome her anorexia, leaving her without friends or hobbies. She suffers from the physical symptoms, including general muscle weakness and loss in bone density, saying that she would rather die than undergo further treatment and wishes to be left in peace. She does not want to be forced into eating anymore. Two experts have declared that the patient has decision-making capacity to refuse further treatment, with consequent risk of dying.	
Case 2: 33-year-old male with schizophrenia, onset at age 17, no significant comorbidities	
Positive symptoms: auditory and visual hallucinations, persecutory delusions. Negative symptoms: apathy, social withdrawal, poverty of speech (all rated severe). Despite long-lasting, high-dose pharmacological treatment (several atypical neuroleptics, haloperidol, clozapine and combinations of these), as well as electro-convulsive therapy, the patient has never been free from positive or negative symptoms. Multiple psychotherapies of various kinds have also failed to stabilize the patient or to improve his quality of life. He does not wish to continue assertive community treatment because he feels it is too intrusive. While the positive symptoms were more dominant in the first years following initial diagnosis, he went on to develop severe negative symptoms, as well as aggression and self-injurious behavior such as burning himself with cigarettes. The negative symptoms and his strong functional deficits are exacerbated by chronic unemployment and inability to live independently, and the patient has no family system. His persisting illness has left him completely isolated, with no social contacts and no hobbies or interests. Two experts have declared that he possesses decision-making capacity in respect of his illness and its treatment.	
Case 3: 40-year-old male with major depressive disorder, no significant comorbidities	
Symptoms: energy loss, insomnia, fatigue, persistent suicidal ideation over 20 years, current acute and concrete suicidal intent. The patient underwent different intensive, evidence-based, long-term psychotherapies, including specialized treatment approaches such as CBASP and IPT. His depression was not improved either by psychotherapy alone or in combination with adequate treatment trials of antidepressants (selective serotonin reuptake inhibitors, tricyclic antidepressants, venlafaxine, augmentation with lithium and antipsychotic medications (quetiapine and aripiprazole)). The patient experienced significant adverse effects with several of the medications. Exhausted and as a last resort, he has decided to undergo electro-convulsive therapy. However, maintenance electro- convulsive therapy proved equally ineffective in preventing the reappearance of suicidal ideation; indeed, the symptoms worsened. The patient experiences severe hopelessness and states that his quality of life is very poor, that he doesn’t want to deal with his illness anymore, and that he plans to commit suicide in the near future. Two experts have declared that he possesses decision-making capacity regarding his illness and its treatment.

**Table 2:** Survey items

I: Questions on the treatment of patients with severe and persistent mental illness (SPMI)	
In the treatment of patients with severe and persistent mental illness (SPMI), how important is:	
A) curing the illness	
B) reduction of suffering	
C) the patient’s ability to function in daily life	
D) the patient remaining autonomous in their decision making	
E) impeding suicide	
According to the World Health Organization (WHO), palliative care ‘is an approach that improves the quality of life of patients and their families facing the problem associated with life-threatening illness, through the prevention and relief of suffering by means of early identification and impeccable assessment and treatment of pain and other problems, physical, psychosocial and spiritual’.	
How strongly do you agree or disagree with the following.	
F) For me, the term ‘palliative’ relates directly to end of life.	
G) For some SPMI patients, palliative care is indicated.	
H) In psychiatry, applying a palliative care model is important in providing optimal support for certain patients without a life-limiting medical illness.	
I) In severe, chronic and therapy-refractory anorexia nervosa, a palliative approach would be suitable.	
J) In severe, chronic and therapy-refractory schizophrenia, a palliative approach would be suitable.	
K) In severe, chronic and therapy-refractory depression, a palliative approach would be suitable.	
L) In severe, chronic and therapy-refractory bipolar disorder, a palliative approach would be suitable.	
M) In severe, chronic and therapy-refractory substance disorder, a palliative approach would be suitable.	
How strongly do you agree or disagree with the following.	
N) SPMI can be a terminal illness.	
O) Sedation for the reduction of unbearable refractory psychological symptoms is justifiable in certain cases of SPMI.	
P) I would generally be willing to perform sedation as mentioned above in ‘O’.	
Q) I generally advocate access to assisted suicide for patients with SPMI.	
R) If physician-assisted suicide was legally permitted for SPMI, I would support my patients in seeking this intervention as the physician of record or by referring them to another physician.	
II: Questions about the three case vignettes^a^	
Please evaluate the case vignettes as below.	
S) I would not be surprised if this patient died within the next 6 months.	
T) For this patient, further interventions to cure the anorexia would most likely be futile.	
U) In this case, I would be comfortable with a reduction of life expectancy in order to increase or maintain quality of life if consistent with the patient’s goals.	
V) In this case, I would accept a temporary decrease in quality of life due to coercive measures.	
W) In this case, I would not proceed against the patient’s wishes.	
X) In this case, sedation to reduce an unbearable refractory symptom is reasonable.	
Y) If physician-assisted suicide was legally permitted, I would support this patient if this was her explicit and enduring wish, referring her to appropriate care.	

^a^Note: Questions S–Y applied to all three case vignettes in Table 1

To examine how psychiatrists would evaluate a patient’s life expectancy, the case vignettes included a ‘surprise question’ (‘I would not be surprised if this patient died within the next 6 months’; see item S in Table 2). In palliative care, variants of the surprise question are often used for patient prognosis near the end of life [24]. The survey included items concerning attitudes to palliative sedation and physician-assisted dying for SPMI patients (see items O, P, Q, R, X and Y in Table 2). These are reported in a separate article.

### Statistical analysis

Arithmetic means were calculated for age and work experience, and descriptive statistics (percentages) were calculated for gender, as well as for all Likert scale items. In order to facilitate readability of the results, Likert scale items were collapsed into three categories (1, 2, 3 = agree, 0 = neutral, − 1, − 2, − 3 = disagree) in the running text.

We included 100% of participants and used available-case analysis, i.e., we indicated the number of missing cases separately for every question, due to the low number of missing cases.

## Results

The survey was mailed to 1311 active members of the SSPP, and 457 surveys (34.9%) were returned of which 85% were fully completed. Of the respondents, 58.8% were male, and 4.2% did not indicate their gender. This gender distribution reflected the total sample of active SSPP members (62.9% male vs. 37.1% female). Mean age was 57.8 years (SD = .43; 95% confidence interval [CI] = 56.9, 58.6; range 35–88, missing *n* = 20), and mean work experience was 27.7 years (SD = .44; CI = 26.8, 28.6; missing *n* = 23).

### Views on the general goals of care in severe and persistent mental illness

In relation to treatment goals for SPMI, respondents most frequently rated reduction of suffering as either important or very important (ratings of 5 or 6 in 94.1% of responses; CI = 91.5, 95.9%; missing *n* = 1). This was followed by daily life functioning (ratings of 5 or 6 in 90.8% of responses; CI = 87.8, 93.1%; missing *n* = 1), autonomy (ratings of 5 or 6 in 76.0% of responses; CI = 71.9, 79.7%; missing *n* = 3), and impeding suicide (ratings of 5 or 6 in 66.1% of responses; CI = 61.6, 70.3%; missing *n* = 3). Only 11.0% of respondents rated curing the illness as an important goal (ratings of 5 or 6; CI = 8.4, 14.2%); a further 49.4% considered this moderately important (ratings of 3 or 4; CI = 44.8, 54.1%; missing *n* = 10).

### Views on palliative care and its use in patients with severe and persistent mental illness

For 45.4% of respondents, the term ‘palliative’ related directly to end of life (ratings of 1, 2 or 3; CI = 40.8, 50.0%); 21.2% remained neutral (rating 0; CI = 17.7, 25.2%), and 33.4% did not relate the term to end of life (ratings of − 1, − 2 or − 3; CI = 29.2, 37.9%; missing *n* = 5). While 78.2% (CI = 74.1, 81.8%) of respondents said that palliative care approaches were indicated for certain SPMI, 11.2% (CI = 86.3, 14.5%) remained neutral (missing *n* = 12). Similarly, 75.8% (CI = 71.6, 79.5%) of respondents thought that application of a palliative care model was important in providing optimal support for certain patients without a life-limiting illness, and 13.6% (CI = 10.7, 17.0%) remained neutral (missing *n* = 7). However, 94.5% (CI = 92.0, 96.2%) of all respondents believed that SPMI could be terminal while 4.4% (CI = 2.9, 6.7%) remained neutral (missing *n* = 4).

When asked about the application of palliative care approaches to different mental disorders, respondents found this most suitable for severe, chronic and therapy-refractory schizophrenia (76.28 rating 1, 2 or 3; CI = 72.8, 80.5%; missing *n* = 4) and for substance disorders (75.3% rating 1, 2 or 3; CI = 71.1, 79.0%; missing *n* = 4). Palliative care approaches were found almost equally suitable for chronic and therapy refractory depression (70.4.7% rating 1, 2 or 3; CI = 66.1, 74.4%; missing *n* = 4); bipolar disorder (67.8% rating 1, 2 or 3; CI = 63.3, 72.0%; missing *n* = 4); and anorexia nervosa (67.7% rating 1, 2 or 3; CI = 63.3, 71.9%; missing *n* = 5) (Fig. 1).

### Responses to vignettes

#### Anorexia nervosa

A majority of respondents indicated that they would not be surprised if the anorexia nervosa patient died within the next 6 months (87.2% rating 1, 2 or 3, where 3 designated *strongly agree*; CI = 83.8, 90.0%; missing *n* = 3). (For all case vignettes, see Table 1). Most respondents agreed that further interventions to cure the anorexia nervosa would most likely be futile (73.1% rating 1, 2 or 3; CI = 68.8, 77.0%; missing *n* = 4), and 82.3% (CI = 78.5, 85.5%) indicated that they would be comfortable with a reduction in life expectancy in order to increase or maintain quality of life in such cases (missing *n* = 5).

#### Schizophrenia

While almost half of respondents indicated that they would not be surprised if the patient diagnosed with schizophrenia died within the next 6 months (45.1%; CI = 40.9, 49.3%), more than a quarter remained neutral (26.9% rating 0; CI = 23.0, 31.2%) with regard to this item (missing *n* = 7). A majority agreed that further interventions to cure the schizophrenia would most likely be futile (64.8% rating either 1, 2 or 3; CI = 60.2, 69.0%; missing *n* = 9), and 72.8% (CI = 68.5, 76.7%) indicated that they would be comfortable with a reduction of life expectancy in order to increase or maintain quality of life (missing *n* = 9).

#### Major depressive disorder

**Fig. 2:**
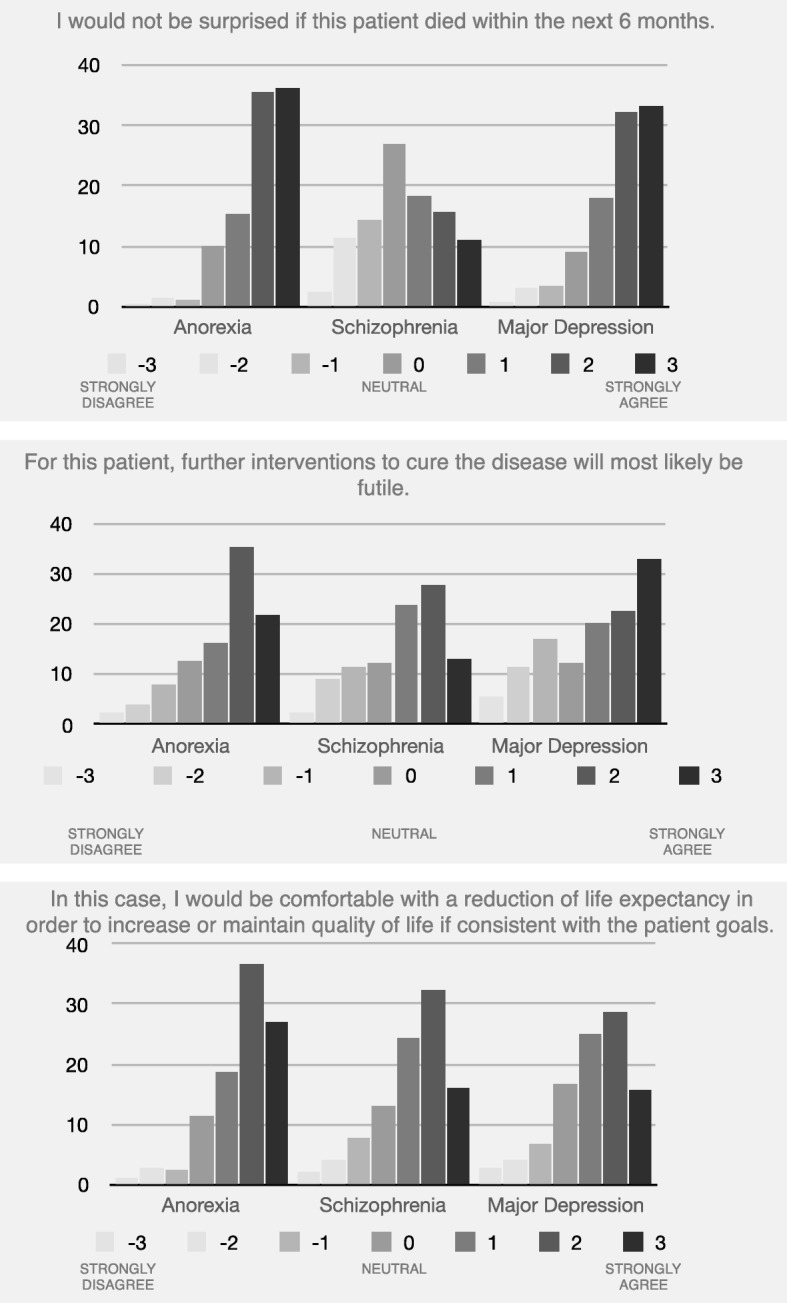
Summary of psychiatrists’ attitudes to life expectancy, futility and quality of life with regard to the three clinical case vignettes

A sizeable majority of respondents indicated that they would not be surprised if the patient diagnosed with major depressive disorder died within the next 6 months (83.8% rating 1, 2 or 3, where 3 designated *strongly agree*; CI = 80.1, 86.9%; missing *n* = 7). Slightly more than half agreed that further interventions to cure the depression would most likely be futile (53.6% rating 1, 2 or 3; CI = 48.9, 58.1%; missing *n* = 7), and 69.3% (CI = 64.9, 73.4%) indicated that they would be comfortable with a reduction of life expectancy in order to increase or maintain quality of life (missing *n* = 7) (Fig. 2).

## Discussion

### Acceptability of palliative care approaches in mental health care

In this survey of psychiatrists in Switzerland, almost all respondents believed that SPMI can be a terminal illness, and that *curing* the illness has a lower priority than other care goals such as *reduction of suffering* and *functioning in daily life*. These findings align with previous conceptual work which suggested that some existing clinical approaches in contemporary psychiatry can be considered palliative because their primary aim is not remission or illness modification [2]. The broad consensus about the fatality of certain severe cases of mental illness is particularly noteworthy given the ‘loud silence’ with regard to death and dying (other than suicide and its prevention) in mental healthcare. Premature mortality is a neglected aspect in mental health care. Accepting it as an unchangeable outcome [25], or completely ignoring it in the development of new treatment approaches is harmful to the most vulnerable of all patients. It is hoped that by acknowledging that this group of patients is at greater risk of dying [26], additional resources can be freed up in order to improve the care of these patients. In summary, our findings suggest widespread agreement among the respondents on the suitability of general palliative care approaches in treating SPMI. One issue raised by several participants in the comment section of the survey was the concern expressed by some experts about the possible impact of characterising mental health treatments as ‘palliative’, which might be seen to imply ‘giving up’ on patients [17–19, 27]. Indeed, the term ‘palliative’ may not be ideal, given its associations with terminal illness [3]; almost half of our respondents felt that it was closely related to end-of-life care, indicating a heterogeneous understanding of palliative care, even among health care professionals.

It is important to stress that the use of palliative care in psychiatry (as in other areas of healthcare) does not exclude other treatment approaches. The features of palliative care approaches, such as the ongoing alliance with patients and their relatives [3], exquisite symptom management and pursuit of patient and family goals for care and for life in general are, for example, compatible and consistent with the principles of the recovery model [4]. In this sense, palliative care approaches may offer psychiatrists additional tools in the care of SPMI, particularly where patient needs and goals cannot be met by current psychiatric interventions. However, the discomfort about introducing approaches that used to be reserved for a terminally ill population in psychiatric treatment of SPMI patients has to be taken seriously and has to be critically accompanied scientifically. Specifically, it has to be evaluated whether using a less loaded term such as *supportive care* can improve acceptance of the concept [28].

With regard to the case vignettes, participants prioritized quality of life over patients’ remaining life expectancy, and in all three cases, the great majority doubted that further interventions to cure the illness would be successful. This finding aligns with conceptual questions concerning the importance of *curing* SPMI as compared to other goals of care such as *reduction of suffering* and *functioning in daily life*. The overall consensus that curative approaches would most likely be futile in certain specific cases of SPMI confirms the need to further explore the concept of medical futility in psychiatry. Objections to the applicability of this concept in cases of chronic psychiatric illness are multifaceted [17, 19]. However, even if one accepts these arguments and concludes that the concept is not relevant in the context of mental illness, the question remains of how best to deal with the reality of unremitting or progressively declining mental illness. In summary, while it could be argued that the recovery-based model and the harm reduction approaches already seek to minimize symptoms in acute and maintenance phases, we believe that the considerations above have implications that go beyond of what is currently available, especially with regard to futility and last resort therapeutic interventions.

### Lack of specificity of the conceptual framework

It seems clear that the concepts and framework underpinning palliative care approaches in a psychiatric context require further elucidation, including the issue of how specific palliative care interventions might be implemented. In particular, advocates need to elaborate how palliative care might be applied to psychiatric illness, including the prevention and relief of suffering, prevention of futile and burdensome interventions and improvement of quality of life. Any such investigation of the feasibility of specific palliative care interventions lies beyond the scope of the present study. It should also be noted that the WHO definition of palliative care provided in the survey (see Table 2) was described by several respondents as vague and applicable to many (if not all) forms of psychiatric treatment. The high variability in the results might be one indication for a lack of consensus on what palliative care approaches in this context would comprise. This aspect hast to be taken into consideration when interpreting the data, and it will be crucial to develop a minimum consensus regarding the definition of palliative care approaches in order to further develop this area.

### Strengths and limitations

The present study has a number of strengths. Previously, palliative care approaches in psychiatry have been discussed mainly at a conceptual level by ethicists and experienced clinicians. We are aware of only one other study that tackles the topic empirically through qualitative interviews [29], focusing on commonalities between contemporary mental healthcare and palliative care philosophies. It is argued here that the similarities between mental health services and palliative care principles can serve as a foundation for integrating these approaches into mental health services.

The present study is the first survey to use quantitative methods to explore mental health professionals’ attitudes to the treatment of SPMI patients in general, and to the use of certain palliative care approaches in particular. In light of the controversy surrounding that discussion, this insight is an important first step towards establishing the relevance of the palliative care concept in mental health care.

The study also has several limitations. Although Likert scale items are an accepted means of conducting quantitative surveys, the options offered can only approximate complex multidimensional concepts.

In assembling the study’s advisory group, our rationale was to include on the one hand persons who were able to advise us with relevant knowledge on the research questions and the content of the survey including experts and trainees in psychiatry, psychology, and palliative care, and on the other hand, professionals with regard to survey design issues and statistics (psychologist and biostatistician). However, it’s a limitation that no other stakeholders such as patients, family, or policy makers have been part of the advisory board. The present evidence depends on only 457 completed surveys. This corresponds to about 10% of psychiatrists in Switzerland. In addition, the sample was confined to German-speaking members of the SSPP and may not be representative for all psychiatrists practicing in Switzerland. Furthermore, the results are not generalizable to other mental health care professionals who might be involved in the care of patients with SPMI such as nurses and psychologists. The nonresponse rate raises the possibility of response bias. It might be possible that psychiatrists with particular pre-existing normative beliefs were disproportionally represented. However, the demographics correspond to the total of all psychiatrists in Switzerland and the results have a high variability, suggesting a minor impact of the response bias on our data. Lastly, it is important to note that an available case analysis was used in order to minimize loss of data.

Lastly, it is important to note that an available case analysis was used in order to minimize loss of data but that the known disadvantages of this, e.g., that the standard of errors computed by most software packages uses the average sample size across analyses, do not apply for our study because we haven’t used inferential statistics and used available cases for SD’s and confidence intervals as well.

### Implications for clinical practice

The present findings indicate that many psychiatrists—at least in Switzerland—consider that palliative care approaches may be suitable for certain cases of SPMI. For clinical practice, this means that palliative care represents a possible option in the treatment of SPMI patients and the psychiatric profession’s readiness to introduce some of these tools to clinical care. To explore this option, the psychiatric profession must design a framework for use and a common language for the field, and must subsequently create an evidence base capturing the impact on clinical outcomes for SPMI patients. Most importantly, palliative approaches must be seen as an addition to rather than a replacement for other novel and promising person-centred approaches, such as the recovery movement [4, 21]. It remains open whether the term ‘palliative’ will have majority appeal or whether palliative care principles will merely inform a modern concept of psychosocial support for SPMI patients.

### Future research

While this study offers some insight into how the surveyed psychiatrists appraise the implementation of palliative care approaches in mental healthcare, it is mainly to be interpreted as a starting point of the discussion. It remains unclear how the concept might be assessed by affected patients, and the specifics of palliative care interventions remain to be defined. The next step will be to develop a framework for differential indication—that is, to identify which patients would qualify for or benefit from a palliative care approach. It will be crucial for further development to adequately involve patients and put their needs first.

## Abbreviations

CEBES: Checklist for the ethical evaluation of empirical studies that don’t need mandatory authorization
IRB: Institutional Review Board
SPMI: Severe and Persistent Mental Illness
SSPP: Swiss Society for Psychiatry and Psychotherapy
WHO: World Health Organization
